# Effectively Controlled Structures of Si-C Composites from Rice Husk for Oxygen Evolution Catalyst

**DOI:** 10.3390/molecules28166117

**Published:** 2023-08-18

**Authors:** Changwei Li, Xin Zhao, Min Gao, Fangong Kong, Honglei Chen

**Affiliations:** State Key Laboratory of Biobased Material & Green Papermaking, Qilu University of Technology, Shandong Academy of Sciences, Jinan 250353, China; lichangwei0205@126.com (C.L.); shdgaomin@whu.edu.cn (M.G.); kfgwsj1566@163.com (F.K.)

**Keywords:** rice husk, Si-carbon material, SiC, controlled, oxygen evolution catalyst

## Abstract

This work explores a simple way to regulate the morphology and structure of biomass-based carbon and effectively utilize its internal functional groups as the substrate for the next energy materials. The unique randomly oriented and highly interconnected cordyceps-like 3D structure of rice husk is formed by direct high-temperature carbonization, and the main component is SiC. The well-arranged cordyceps-like structure of SiC demonstrates a remarkable structural/chemical stability and a high rate of electron migration, and further could be used as a stable substrate for metal deposition and find application in the field of electrocatalysis. The oxygen evolution reaction catalyst (SiC-C@Fe_3_O_4_) prepared by chemical deposition exhibits a low overpotential (260 mV), low Tafel slope (56.93 mV dec^−1^), high electrochemical active surface area (54.92 mF cm^−2^), and low Rct value (0.15 Ω) at a current density of 10 mA cm^−2^ in 1 M KOH electrolyte. The produced natural Si-C composite materials overcome the limitations imposed by the intricate internal structure of silicon-rich biomass. The existence of this stable substrate offers a novel avenue for maximizing the utilization of rice-husk-based carbon, and broadens its application field. At the same time, it also provides a theoretical basis for the use of rice husks in the field of hydrogen production by electrolysis of water, thus promoting their high-value utilization.

## 1. Introduction

The electrocatalytic strategy for water splitting is widely regarded as one of the most feasible methods for producing zero-carbon hydrogen (H_2_) and environmentally friendly oxygen (O_2_) [1,2,3]. The oxygen evolution reaction (OER), as a crucial electrode reaction in water splitting for hydrogen production, involves a four-electron transfer process. Its slow kinetic reaction and the constraints posed by expensive and scarce precious-metal-based catalysts have hindered the profound advancement of water splitting technology [4,5]. Nonprecious metal catalysts are promising and effective materials expected to replace commercial precious-metal-based catalysts [6,7]. However, due to harsh reaction environments, nonprecious-metal-based electrocatalysts in the pure metal state usually do not survive directly in acidic and alkaline electrolytes; conjugation with a stabilized substrate is an effective way to alleviate this problem [8,9].

Carbon materials, as a substrate for metal loading, not only provide excellent electrical conductivity, a high number of active sites, and chemical stability but also enhance the overall activity and tunability of materials, offering strong support for efficient, cost-effective, and environmentally friendly electrocatalytic processes [10,11]. Additionally, the organic combination of carbon materials with metals plays a protective role in metal catalysts, prolonging their lifespan and improving the efficiency and stability of catalytic reactions [12]. This is crucial for the widespread application of metal catalysts in the fields of electrochemistry and catalysis. Transition metal (Co, Ni, Fe, Cu) single-atom catalysts anchored on 3D nitrogen-doped porous carbon nanosheets as efficient oxygen reduction electrocatalysts for a Zn-Air battery could achieve a maximum power density of 220 mW cm^−2^ at 368 mA cm^−2^ [13]. An FeCoNi/chitosan gels/NF OER electrocatalyst with a 3D porous network architecture was synthesized by an impregnation-foaming-calcination method and could achieve a current density of 500 mA cm^−2^ at an overpotential of 325 mV [14]. The multi-metal (Zn, Co, Ni)@N-doped carbon bifunctional catalysts were prepared by metal deposition in a N-doped carbon interfacial matrix, which could achieve a current density of 10 mA cm^−2^ at an overpotential of 68 mV for the HER and a current density of 10 mA cm^−2^ at an overpotential of 186 mV for the OER [15].

Further research on high-energy-density carbon-based electrodes and low-energy-barrier metal catalysts using biomass-based carbon as the matrix is an effective way to solve the double carbon problem [16,17]. Biomass is natural, green, and renewable, with abundant pore structures; it serves as an excellent precursor for preparing carbon materials. The effective regulation of biomass-based carbon morphology and pore structure is still an important research direction to expand its application field [18].

The rice husk is the hard protective coating of the grain that separates during hulling. It is a biomass rich in silicon (Si). It is a relatively high-volume, low-cost by-product that contains Si, providing a certain active site on the surface of biomass-based carbon [19,20]. However, the presence of internal Si affects the pore structure of biomass-based carbon, leading to a narrower pore distribution, smaller pore size, and lower porosity of carbon materials [21]. The structure and properties of Si within the husk could be leveraged to regulate the relationship between Si and carbon (C) simply and efficiently, using the rich Si content present in the rice husk as a starting point, enabling the preparation of a rice-husk-based Si-C material with controllable morphology and stability. Silicon carbide (SiC) has a diverse set of favorable mechanical, thermal, chemical, and electrical properties, making it a highly sought-after non-oxide ceramic material in numerous engineering applications. The preparation of natural SiC-C composites from renewable rice husks provides a strategy for sustainable development. At the same time, it also provides an effective way to achieve the goal of double carbon. Ultrafine SiC is prepared from rice husks by a heat treated–radio frequency plasma process, which requires high-cost equipment [22]. Rice husks are first pyrolyzed in inert gas, and then electrochemical treatment is performed in molten NaCl-KCl-MgCl_2_ to prepare SiC materials, which complicates the whole process and reduces the treatment efficiency [23]. In addition, SiC can be prepared by the sol–gel process [24], laser pyrolysis [25], or the microwave method [26]. The sol–gel process requires expensive precursor solutions and is a complicated process, while the laser synthesis and microwave synthesis have very high operating costs with expensive equipment. On the other hand, high-temperature carbonization of rice husks is a simple and effective method to prepare SiC-C composites with regular morphology. This material could serve as a valuable stable matrix for new biomass-based carbon applications, providing an essential theoretical foundation for energy storage and conversion. This approach holds significant promise for advancing biomass-based carbon material research and development, and also promotes the realization of high-value utilization of biomass materials.

This study aimed to use rice husk as a raw material and regulate the morphology of carbon within them through high- and ultra-high-temperature carbonization. The objective was to analyze the composition and connection mode of carbon in rice husk, with the aim of effectively constructing a stable and controllable Si-C composite.

## 2. Results and Discussion

Rice husk contains approximately 30–50% organic carbon [27]. It accounts for around 20% of the weight of rice. The components of rice husk have been analyzed. The cellulose content was found to be 50%, while the lignin content was 25–30%. Moreover, the silicon dioxide (SiO_2_) content was between 15% and 20%, and the moisture content typically ranged from 10% to 15% [28]. As rice is an aquatic plant, it absorbs orthosilicic acid (Si(OH)_4_, a water-soluble mono-silicic acid molecule, which combines with its hydrophilic components through intermolecular interaction to generate SiO_2_·nH_2_O). This compound is finally deposited on epidermal cells, cell walls, and cell spaces, which explains why the Si element content is the highest among non-wood raw materials [29].

**Figure 1:**
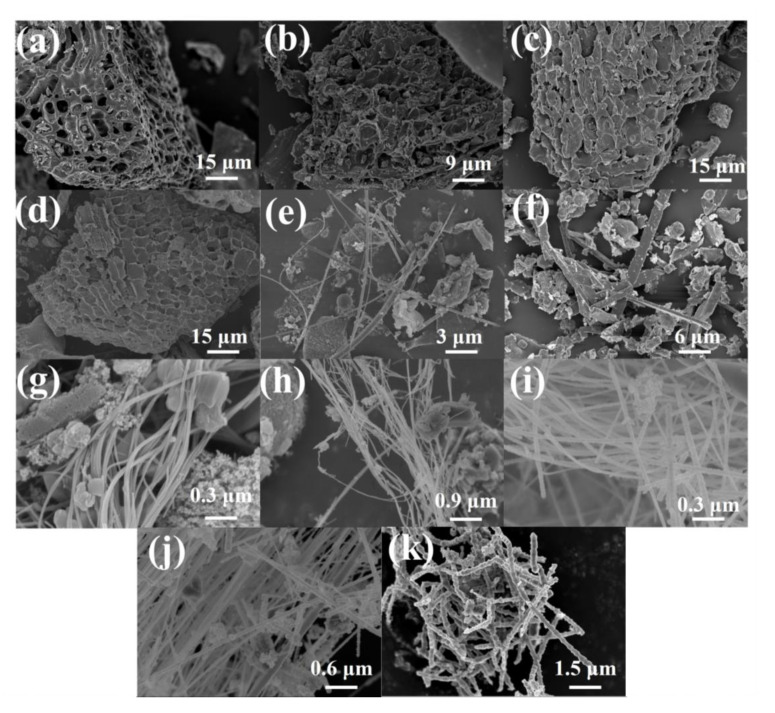
SEM images of RH-1000 (**a**), RH-1100 (**b**), RH-1200 (**c**), RH-1300 (**d**), RH-1400 (**e**), RH-1500 (**f**), RH-1520 (**g**), RH-1540 (**h**), RH-1560 (**i**), RH-1580 (**j**), and RH-1600 (**k**).

The morphologies of rice husk samples after carbonization at different temperatures are assessed using a scanning electron microscope (SEM), and the images are displayed in Figure 1. The RH-1000, RH-1100, RH-1200, and RH-1300 samples exhibit abundant pore structures (Figure 1a–d). However, the pore walls gradually collapse with an increase in carbonization temperature, and the pore structures are destroyed. Interestingly, when the carbonization temperature increases to 1400 °C, 1500 °C, or even 1600 °C, the pore structures in the materials are almost entirely destroyed. For instance, the RH-1400 sample shows a long rod-like structure (Figure 1e), the RH-1500 sample shows a cuboid shape (Figure 1f), and the RH-1600 sample displays a particular cordyceps morphology (Figure 1k). The morphologies of samples after carbonization at 1520 °C, 1540 °C, 1560 °C, and 1580 °C are investigated to further explore the morphological trend at these temperatures (Figure 1g–j). They all exhibit similar morphological characteristics, with long bar structures cross-linked with a few cordyceps structures present among them.

**Figure 2:**
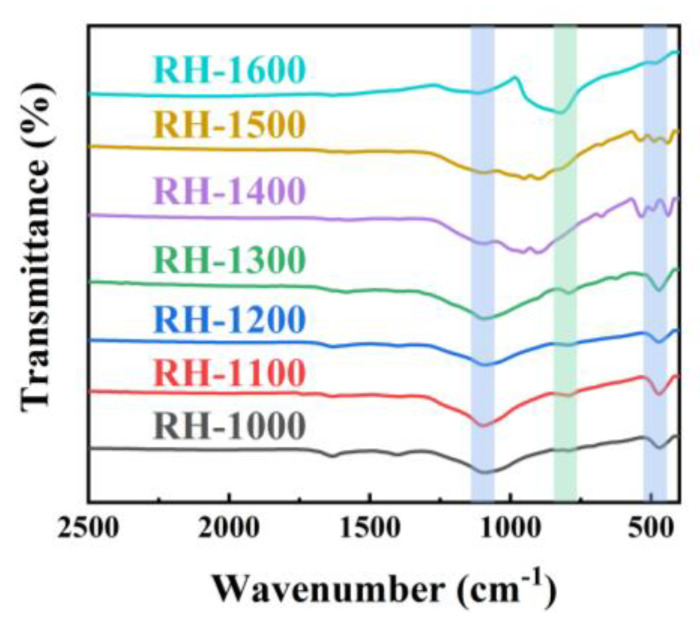
FT-IR spectra of RH-1000, RH-1100, RH-1200, RH-1300, RH-1400, RH-1500, and RH-1600.

The chemical structures of the RH samples, including RH-1000, RH-1100, RH-1200, RH-1300, RH-1400, RH-1500, and RH-1600, were characterized using Fourier transform infrared (FT-IR) spectroscopy to elucidate their composition. As evidenced in Figure 2, the curve trends of the samples are generally consistent, indicating the presence of similar functional groups. The two infrared absorption peaks at 1093.2 cm^−1^ and 475.3 cm^−1^ are related to the stretching vibration of Si-O-Si, and the additional absorption peaks at 828.1 cm^−1^ correspond to Si-C groups, indicating that the Si element in carbonized rice husk exists in the form of Si-O-Si and Si-C groups [30]. However, the intensity variation in the absorption peaks corresponding to the two groups is opposite, which may be attributed to the occurrence of the following reactions [31]:

SiO_2_ + 3C = SiC + 2CO (1)

With the progress of the reaction, SiO_2_ is gradually consumed, resulting in a gradual decrease in the peak intensity of the Si-O-Si groups, and, in contrast, the peak intensity of the Si-C groups gradually increases. Higher carbonization temperatures promote the occurrence of the reaction, so the maximum SiC content is achieved at a carbonization temperature of 1600 °C.

The synthesis of SiC through the carbothermal reduction of silica is considered a multi-stage process involving vapor–solid growth mechanisms and a series of chemical reactions that lead to the formation of either particles or whiskers. Because of the intimate contact available between carbon and silica in rice husk, SiC is formed at a relatively lower temperature [32]. The reaction is a gas–solid interaction between SiO and C:

SiO_2_ + C = SiO + CO (2)

SiO + 2C = SiC + CO(3)

SiC is over-generated and deposited due to the continuous reaction, forming SiC crystal nuclei and crystal particles. When SiO_2_ and carbon are gradually consumed by Reaction (2), they no longer remain in contact. Then, CO produced by Reaction (2) reacts with SiO_2_ to form SiO and CO_2_.

SiO_2_ + CO = SiO + CO_2_ (4)

At higher carbonization temperatures, SiO and CO react as follows, which is a gas–gas interaction [33]:

SiO + 3CO = SiC + 2CO_2_ (5)

SiC continues to be deposited and grow in its original direction, leading to the formation of whiskers. However, crystal defects (e.g., dislocations and stacking faults) occur during the SiC growth, resulting in significant surface irregularities and a cordyceps-like structure [34,35].

**Figure 3:**
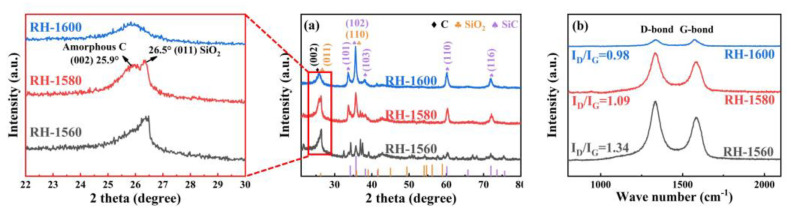
(**a**) XRD pattern and (**b**) Raman spectra of RH-1560, RH-1580, and RH-1600.

The X-ray diffraction (XRD) patterns of the RH-1560, RH-1580, and RH-1600 samples are depicted in Figure 3a. As can be seen in the partially enlarged view of the 20–30° range, the sharp diffraction peak at 26.5° corresponds to the (011) crystal face of SiO_2_ (JCPDS card no. 47-1144). While the diffusion scattering peak at 25.9° is related to the (002) crystal face of the carbon materials, confirming the amorphous characteristics of the samples [36,37,38,39]. Moreover, other diffraction peaks of the sample correspond to hexagonal SiC (JCPDS card no. 29-1131) with space group P63mc (186), indicating the presence of SiC [40].

The degree of defects in the samples was further studied using Raman spectroscopy. As shown in Figure 3b, the D band (1340 cm^−1^) corresponds to the disordered or defective structure in the carbon materials, while the G band (1580 cm^−1^) represents the ordered graphitic carbon structure in carbon materials [41]. Additionally, the I_D_/I_G_ ratios of the RH-1560, RH-1580, and RH-1600 samples are 1.34, 1.09, and 0.98, respectively. The ratios exhibit a decreasing trend, which is attributed to the gradual accumulation of regular SiC in the samples as the temperature increased, resulting in an increasing ordering degree of the samples.

**Figure 4:**
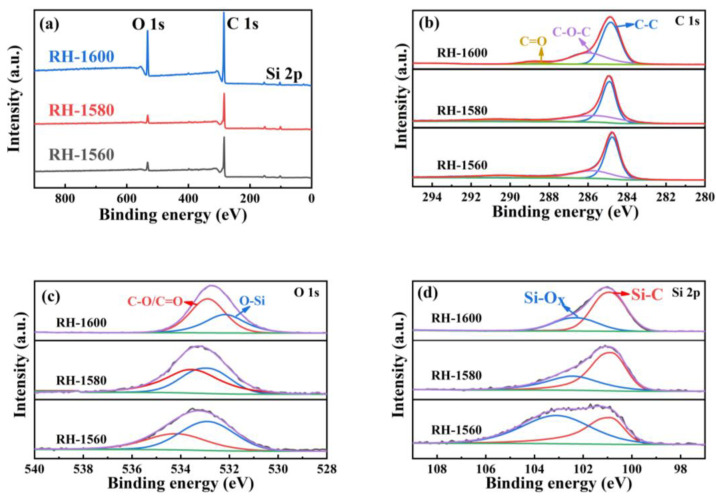
(**a**) XPS patterns of RH-1560, RH-1580, and RH-1600 and high-resolution spectra of (**b**) C 1s, (**c**) O 1s, and (**d**) Si 2p of RH-1560, RH-1580, and RH-1600.

Figure 4a represents the X-ray photoelectron spectroscopy (XPS) full spectrum of the sample, containing C, O, Si, and other elements. The high-resolution spectral diagrams of the RH-1560, RH-1580, and RH-1600 samples are fitted to segregate peaks, and the spectral diagrams of C1s (Figure 4b) display three Gaussian peaks corresponding to C-C (284.7 eV), C-O-C (285.8 eV), and C=O (288.1 eV), respectively. As shown in Figure 4c, the O-1s core XPS spectra of the RH-1560, RH-1580, and RH-1600 samples display two Gaussian peaks corresponding to O-Si and C-O/C=O groups, respectively. The binding energy position of these peaks is shifted, which may be due to the SiO_2_ + 3C = SiC + 2CO reaction occurring during the high-temperature carbonization process. Furthermore, the area ratio of O-Si and C-O/C=O bonds decreases, which is related to the consumption of SiO_2_ as a reactant [42]. As can be seen from Figure 4d, the XPS spectra of the Si 2p core level illustrate two Gaussian peaks: Si-C (100.9 eV) and Si-O_x_ (102.5 eV). With the generation of SiC and the consumption of SiO_2_, the area ratio of the Si-C and Si-O_x_ bonds gradually increases. This may be due to the higher carbonization temperature, which facilitates the reaction [43]. Combined with the FTIR and XRD patterns, the XPS spectra demonstrate the presence of SiC in the material [44].

**Figure 5:**
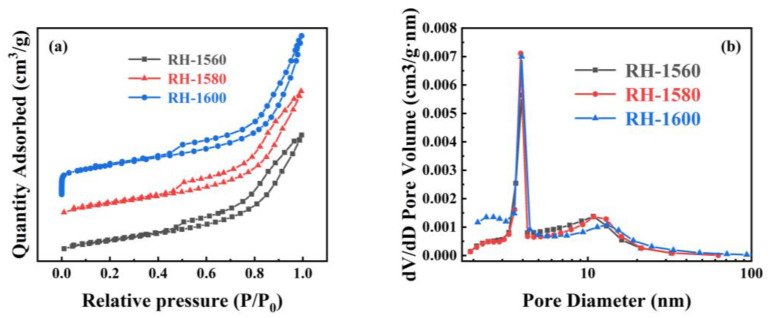
(**a**) N_2_ adsorption and desorption isothermal curves and (**b**) pore size distribution curves of RH-1560, RH-1580, and RH-1600.

The N_2_ absorption and desorption curves, along with the pore size distribution curves, of the RH-1560, RH-1580, and RH-1600 samples are shown in Figure 5. As shown in Figure 5a, all samples exhibit type IV isotherms and H3 hysteresis rings when the relative pressure (*P*/*P*_0_) ranges from 0.1 to 1.0, indicating the presence of mesopore and micropore structures [45]. The pore size distribution diagram reveals that the pore sizes of the samples are mainly distributed in the ranges of 2–4 nm and 10–15 nm (Figure 5b). The respective BET specific surface areas of the RH-1560, RH-1580, and RH-1600 samples are 100.2, 107.4, and 121.9 m^2^ g^−1^, respectively (Table 1). Compared with conventional biomass-based carbon materials, their specific surface areas are relatively low, likely due to a higher mesopore content and a lower micropore content, as indicated by their micro–mesopore ratio. This effect is attributed to the presence of Si within the Si-C material, which could impact the pore structure and porosity of biomass-based carbon [46]. Consequently, their electrochemical storage capacity is limited. The samples also exhibit pore volumes of 0.191, 0.205, and 0.278 cm^3^ g^−1^ and average pore sizes of 8.2, 8.7, and 9.1 nm, respectively. As the carbonization temperature increased, the collapse of the pore structure led to an increase in mesopores and macropores. This resulted in the destruction of the pore structure, causing a gradual increase in both the pore volumes and average pore diameters of the samples.

**Figure 6:**
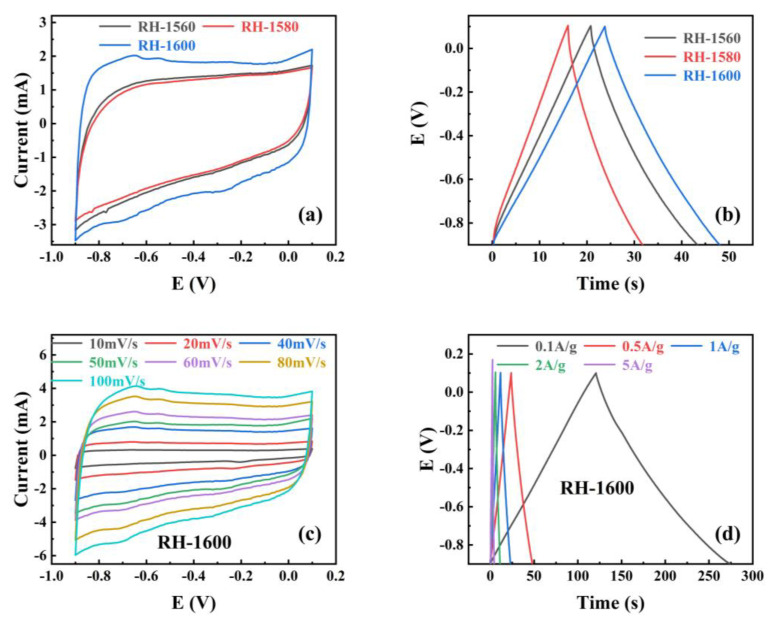
(**a**) Cyclic voltammograms curves (CV) at 50 mV s^−1^ scan rates for different samples; (**b**) galvanostatic charge–discharge (GCD) of different samples at 0.5 A g^−1^; (**c**) the CV of RH-1600 sample from 10 to 100 mV s^−1^ scan rate, (**d**) the GCD of RH-1600 sample from 0.1 to 5 A g^−1^.

To investigate the performance of RH samples as electrode materials for supercapacitors, the cyclic voltammetry (CV) and galvanostatic charge–discharge (GCD) of the RH-1560, RH-1580, and RH-1600 samples were tested in a three-electrode system with an electrolyte solution of 1.0 M KOH, and the results are shown in Figure 6. Figure 6a shows the cyclic voltammetry (CV) curves obtained for all samples at a scan rate of 50 mV s^−1^. The curves exhibit rectangular-like shapes without distinct oxidation or reduction peaks, indicating that the capacitance of the samples is primarily provided by the electrostatic double-layer capacitor (EDLC) [47]. Furthermore, the enclosed area of the CV curve reflects the capacitance value of the electrode material. Among them, the RH-1600 sample exhibits the largest enclosed area in the CV curve, indicating the highest capacitance value. The galvanostatic charge–discharge (GCD) curves of the samples at a current density of 0.1 A g^−1^ are displayed in Figure 6b. The curves exhibit a triangular shape, resembling an isosceles triangle. Among them, the RH-1600 sample shows the longest discharge time, indicating the highest specific capacitance. The specific capacitance is calculated from the discharge plots by the following equations [48]:

C = IΔt/(mΔV)(6)

where C is specific capacitance (F g^−1^), I is discharge current (A), and ΔV is the potential change within the discharge time Δt (s).

After calculation, the specific capacitances of the RH-1560 and RH-1580 samples are found to be 11.3 and 7.9 F g^−1^ at 0.5 A g^−1^, respectively. The RH-1600 sample exhibits the highest specific capacitance, calculated to be 12.4 F g^−1^ at 0.5 A g^−1^, which is consistent with the results reflected in the CV curves and GCD curves. To further investigate the capacitance performance of the RH-1600 sample, CV curves are obtained at different scan rates (10–100 mV s^−1^), as shown in Figure 6c. The curve shape remains relatively stable as the scan rate increases, indicating a stable structure of the sample [49]. Figure 6d illustrates the galvanostatic charge–discharge (GCD) curves of the RH-1600 sample at various current densities (0.1–5 A g^−1^). The curves exhibit a triangular shape similar to an isosceles triangle, and the longest discharge time corresponds to a current density of 0.1 A g^−1^. Due to the ultra-high temperature carbonization process, the pore structure of the material is seriously damaged, which affects the capacitive performance of the material.

**Figure 7:**
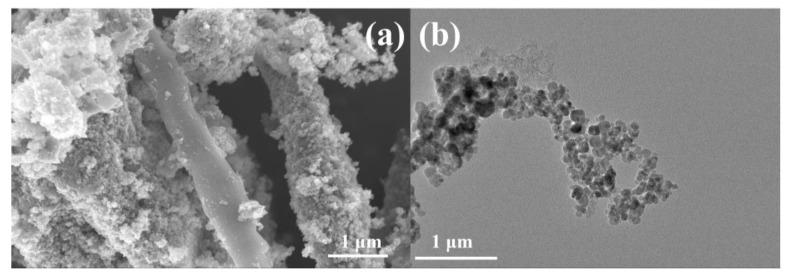
(**a**) SEM and (**b**) TEM images of SiC-C@Fe_3_O_4_ sample.

In order to further explore the applicability of cordyceps-like SiC in the field of electrochemistry, an OER catalyst containing Fe_3_O_4_ nanoparticles was prepared using the metallic chemical deposition–carbonization method using the Si-C cordyceps structure as a matrix. Preliminary implementation of the process resulted in the deposition of Fe_3_O_4_ nanoparticles on the SiC-C@Fe_3_O_4_ sample, forming relatively regular spheres with an average diameter of 15 nm, distributed around the cordyceps structure, as shown in Figure 7a,b. The uniform deposition of the Fe_3_O_4_ nanoparticles could provide a greater active area and promote the occurrence of the oxygen evolution reaction [50].

**Figure 8:**
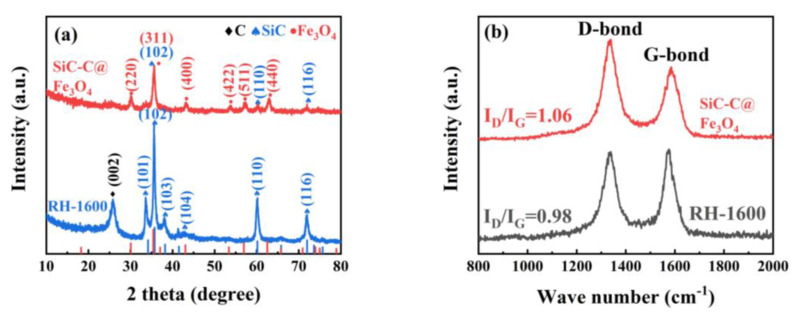
(**a**) XRD patterns and (**b**) Raman spectra images of RH-1600 and SiC-C@Fe_3_O_4_ samples.

XRD patterns of the RH-1600 and SiC-C@Fe_3_O_4_ samples are displayed in Figure 8a. Compared with the RH-1600 sample, the SiC-C@Fe_3_O_4_ sample shows different sharp diffraction peaks. These sharp diffraction peaks belong to the typical cubic phase Fe_3_O_4_ of the space group Fd-3m (227), corresponding to the standard card of Fe_3_O_4_ (JCPDS card no. 19-0629), suggesting the successful deposition of Fe_3_O_4_ nanoparticles [51].

Figure 8b represents the Raman spectra of the RH-1600 and SiC-C@Fe_3_O_4_ samples. The D band (1340 cm^−1^) corresponds to a disordered or defective structure in carbon materials, while the G band (1580 cm^−1^) represents the ordered graphitic carbon structure in carbon materials. According to the I_D_/I_G_ values of the RH-1600 and SiC-C@Fe_3_O_4_ samples, it can be concluded that the defect degree of the SiC-C@Fe_3_O_4_ sample increases after metal deposition. Moreover, these defect structures provide more active sites and promote the production of intermediate ions in the OER [52].

**Figure 9:**
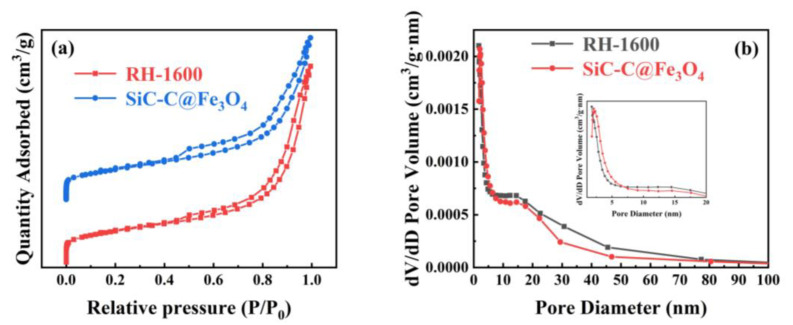
(**a**) N_2_ adsorption and desorption isothermal curves and (**b**) pore size distribution curves of RH-1600 and SiC-C@Fe_3_O_4_ samples.

The N_2_ adsorption and desorption isothermal curves and pore size distribution curves of the samples for RH-1600 and SiC-C@Fe_3_O_4_ are shown in Figure 9. Similar to the isotherm curves of the RH-1600 sample, the SiC-C@Fe_3_O_4_ sample exhibits a type IV isotherm with a type H3 hysteresis ring at a relative pressure (*P*/*P*_0_) ranging from 0.1 to 1.0, indicating the coexistence of micropore and mesopore structures. Moreover, the pore size of the SiC-C@Fe_3_O_4_ sample is mainly distributed in the ranges of 2–4 and 12–17 nm (Figure 9b). After metal deposition, the specific surface area of the sample increases from 121.9 (RH-1600) to 125.5 (SiC-C@Fe_3_O_4_) m^2^ g^−1^ (Table 2). The increase in the specific surface area of the sample may be due to the deposition of Fe_3_O_4_ nanoparticles, which creates a partial pore structure [53]. At the same time, the *S*_meso_/*S*_BET_, pore volume, and average pore size of the SiC-C@Fe_3_O_4_ sample also increase to 83.6%, 0.327 cm^3^ g^−1^, and 10.4 nm, respectively. The high specific surface area and suitable pore size of the SiC-C@Fe_3_O_4_ sample promote ion penetration and mass transfer, and improve the catalytic activity of the OER [54].

**Figure 10:**
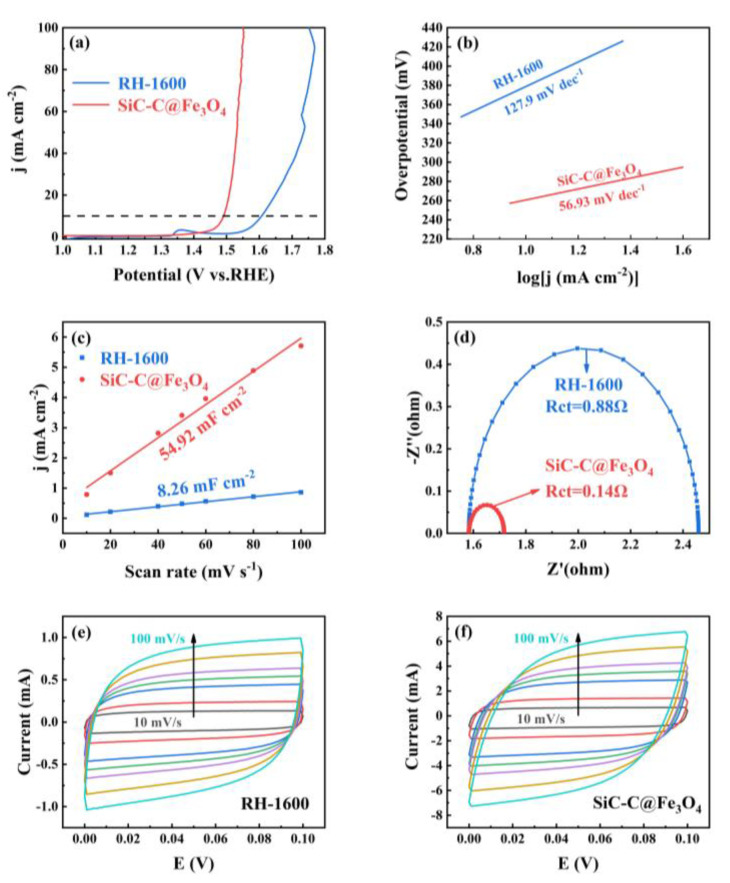
(**a**) LSV curves of RH-1600 and SiC-C@Fe_3_O_4_; (**b**) Tafel plots of RH-1600 and SiC-C@Fe_3_O_4_; (**c**) plots of corresponding current density against scan rate for RH-1600 and SiC-C@Fe_3_O_4_; (**d**) EIS Nyquist plots of RH-1600 and SiC-C@Fe_3_O_4_; (**e**) CV curves of RH-1600 from 10 to 100 mV s^−1^ scan rate; and (**f**) CV curves of SiC-C@Fe_3_O_4_ from 10 to 100 mV s^−1^ scan rate.

The electrocatalytic OER of the RH-1600 and SiC-C@Fe_3_O_4_ samples was evaluated using the standard three-electrode system in 1 M KOH solution. As can be seen from Figure 10a, the RH-1600 sample requires an overpotential of 360 mV to provide a current density of 10 mA cm^−2^, while the SiC-C@Fe_3_O_4_ sample requires 260 mV to achieve a current density of 10 mA cm^−2^, showing excellent catalytic performance. The Tafel slopes of RH-1600 and SiC-C@Fe_3_O_4_ are 127.9 mV dec^−1^ and 56.93 mV dec^−1^, respectively. The smaller Tafel slope of SiC-C@Fe_3_O_4_ indicates that it has better OER kinetic activity. The electrochemical active surface area (ECSA) of the samples was evaluated using the electrochemical double-layer capacitance (C_dl_) proportional to the ECSA. As demonstrated in Figure 10c, the C_dl_ value of SiC-C@Fe_3_O_4_ (54.92 mF cm^−2^) is larger than that of RH-1600 (8.26 mF cm^−2^), which reveals that SiC-C@Fe_3_O_4_ has more active sites, thus promoting the occurrence of the oxygen evolution reaction [55]. Furthermore, EIS was performed to further demonstrate the excellent catalytic performance of the samples. Charge transfer resistance (Rct) at the interface between the sample and the electrolyte solution is indicated by the semicircle observed in the high-frequency region [56]. As detailed in Figure 10d, the Rct value of RH-1600 is 0.88 Ω. Compared with the RH-1600 sample, SiC-C@Fe_3_O_4_ has a small Rct value of 0.14 Ω, indicating that the electron transfer rate of the SiC-C@Fe_3_O_4_ sample is greatly increased after the deposition of Fe_3_O_4_ nanoparticles, thus promoting the transfer of charge in the oxygen evolution reaction. As can be seen in Figure 10e,f, the CV curves obtained for the RH-1600 and SiC-C@Fe_3_O_4_ samples exhibit rectangular-like shapes without distinct oxidation or reduction peaks, indicating that the capacitance in the range of 0–0.1 V is mainly provided by the electrostatic double-layer capacitor. Furthermore, the enclosed areas of the CV curves reflect the capacitance value of the electrode material. SiC-C@Fe_3_O_4_ could reach a larger current range over the same voltage, indicating the excellent activity, which is consistent with the results of ECSA.

The catalytic performance research shows that the as-prepared samples exhibited comparable or even better catalytic performance for the OER in an alkaline solution than other similar materials, with a relatively low overpotential (260 mV) and Tafel slope (56.93 mV dec^−1^). Table 3 shows the catalytic effects of similar materials. The present study focused on the regulation of rice husk, and thus, only presented a preliminary application of the electrocatalytic performance. However, the details of this performance need to be further studied in the future.

## 3. Experimental Method

### 3.1. Materials

#### 3.1.1. Preparation of Rice-Husk-Based Carbon Materials

The rice-husk-based carbon material (RH) was prepared by crushing and sifting rice husks (60 mesh), washing them with deionized water, and drying them at 105 °C. Next, a specific amount of rice husks was carbonized in a high-temperature tube furnace under a protective atmosphere of nitrogen for 2 h at a heating rate of 5 °C min^−1^. The carbonization temperatures used were 1000 °C, 1200 °C, 1300 °C, 1400 °C, 1500 °C, 1520 °C, 1540 °C, 1560 °C, 1580 °C, and 1600 °C. The obtained rice-husk-based carbon materials are expressed here as RH-X, where X is the carbonization temperature. The resulting samples exhibited different properties depending on the carbonization temperature used.

#### 3.1.2. Preparation of Fe_3_O_4_/Rice-Husk-Based Carbon Composites

The Fe_3_O_4_/rice-husk-based carbon composites were prepared by dissolving 5.5 g FeCl_3_·6H_2_O and 3.4 g Fe_2_SO_4_·7H_2_O in 100 mL DI water, followed by the addition of 2 g RH-1600 and 12 mL 25 wt.% NH_3_·H_2_O. The solution mixture was stirred for 2 h at 90 °C. The resulting mixtures were washed with DI water until they reached a neutral pH, and further carbonized at 500 °C for 2 h, at a heating rate of 5 °C min^−1^ under a N_2_ atmosphere. The obtained Fe_3_O_4_-carbon composites were designated as SiC-C@Fe_3_O_4_.

**Figure 11:**
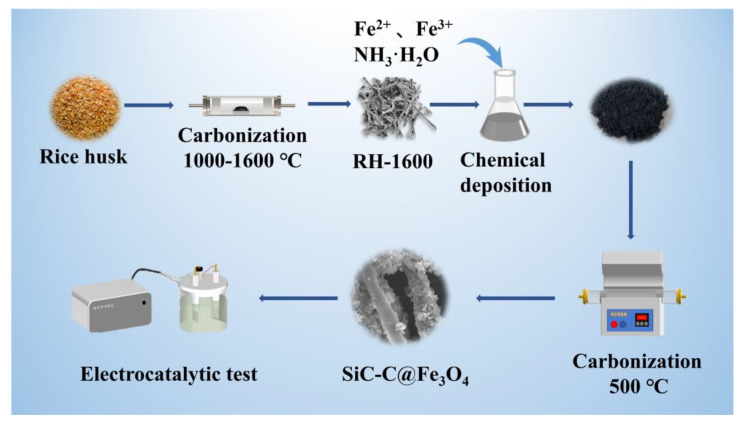
Schematic diagram of sample preparation.

A schematic diagram of the sample preparation is shown in Figure 11.

### 3.2. Characterization

The surface morphology of the samples was examined using an environmental scanning electron microscope (JSM-7401F, Hitachi, Tokyo, Japan) at an accelerating voltage of 10 kV. Nitrogen adsorption–desorption isotherms and pore size distributions were determined using Micromeritics ASAP 2460 (Maike, Smyrna, Georgia, USA). The analysis was conducted using the low-temperature liquid nitrogen (77 K) adsorption method at relative pressures (*P*/*P*_0_) ranging from 1 × 10^−5^ to 1. The Brunauer–Emmett–Teller (BET) method was used to measure the specific surface area, and the pore volume was determined at *P*/*P*_0_ = 0.99. The pore size distribution was established using the density functional theory (DFT) method. The degree of graphitization of the samples was studied using an X-ray diffractometer (D8-ADVANCE, Bruker, Rheinstetten, Germany). The defect degree of the carbon materials was analyzed using laser confocal Raman spectroscopy (InVia reflex, Renishaw inVia, London, UK). The chemical bonds in the samples were investigated using X-ray photoelectron spectroscopy (ESCALABXi+, Thermo Fisher Scientific, Waltham, MA, USA). The electrochemical properties of the carbon materials were investigated using an electrochemical workstation (IVIUM, Eindhoven, The Netherlands).

### 3.3. Electrochemical Test

The electrocatalytic performance of each sample was evaluated using the CHI-660E electrochemical workstation (CHI Instruments, Shanghai, China) with 1 M potassium hydroxide (KOH) solution as the electrolyte. A nickel foam working electrode with a surface area of 1 cm^2^ was used, and the loading was a composition comprising 80 wt.% of active material (biochar composite ball milling for 24 h), 10 wt.% of conductive agent (carbon black), and 10 wt.% of binder (PTFE powder). The reference electrode and the opposite electrode used were Ag/AgCl electrodes filled with graphite rods and a saturated KCl solution, respectively. The prepared sample was mixed with carbon black and polyvinyl fluoride at a ratio of 8:1:1, followed by the addition of an appropriate amount of pyrrolidone solution. The mixture was ultrasonically treated for 15 min to achieve uniform mixing. The resulting mixture was evenly applied onto a nickel sheet, dried at 105 °C for 12 h, and pressed under 6 MPa pressure using a tablet press for 30 s. High-purity O_2_ was introduced into the KOH solution for 30 min before the electrochemical measurement.

Subsequently, the electrode was prepared by cyclic voltammetry (CV) and scanned to stabilize the electrode current (from 0 to 1 V, 200 cycles). The polarization curves of the linear sweep voltammetry (LSV) measurements were acquired via ohmic potential drop (iR) correction in 1 M KOH solution at a scan rate of 10 mV s^−1^. The CV tests were conducted from 0 to 0.1 V (vs. RHE) at different scan rates (from 10 to 100 mV s^−1^) to determine the electrochemical double-layer capacitance (C_dl_). Electrochemical impedance spectroscopy (EIS) studies were performed within the frequency range of 10^6^ to 0.01 Hz at an amplitude of 5 mV.

## 4. Conclusions

This study mainly investigated the morphology changes and formation mechanism of rice-husk-based carbon at different carbonization temperatures. At a carbonization temperature of 1600 °C, the successfully prepared SiC shows a regular cordyceps-like morphology. Moreover, it presents a randomly oriented and highly interconnected 3D structure. Compared with other preparation processes such as the molten salt method, sol–gel method, and plasma method, the carbonization process is simpler and more convenient. Although this cordyceps SiC has a regular morphology, its low porosity limits its application in adsorption or double-layer supercapacitors, but this structure is suitable for the deposition and growth of metals or metal oxides. Its electron mobility is high, making it capable of carrying large current densities. Furthermore, it possessed good chemical stability, making it suitable for electrochemical tests. Therefore, Fe_3_O_4_ was deposited on the cordyceps-like SiC matrix, and the electrocatalytic performance of the SiC-C@Fe_3_O_4_ sample was evaluated. Compared with other materials, the as-prepared samples displayed good catalytic performance for the OER in an alkaline solution with a relatively low overpotential (260 mV), low Tafel slope (56.93 mV dec^−1^), high electrochemical active surface area (54.92 mF cm^−2^), and low Rct value (0.15 Ω). The effective regulation of rice husk structures to produce natural Si-C composites overcame the limitations caused by the complex internal structure of Si-rich biomass and demonstrated the efficient use of Si in Si-rich biomass. The application of Si-rich biomass waste in energy storage and conversion was also expanded. The existence of this matrix structure provides a novel approach for further using rice-husk-based carbon, and also promotes the high-value utilization of silicon-containing biomass materials.

## Figures and Tables

**Table 1:** Pore structure parameters of RH-1560, RH-1580, and RH-1600.

Sample	*S*_BET_ (m^2^ g^−1^)	*S*_meso_/*S*_BET_ (%)	Pore Volume (cm^3^ g^−1^)	Average Pore Size (nm)
RH-1560	100.2	91.3	0.191	8.2
RH-1580	107.4	88.9	0.205	8.7
RH-1600	121.9	83.5	0.278	9.1

**Table 2:** Pore structure parameters of RH-1600 and SiC-C@Fe_3_O_4_ samples.

Sample	*S*_BET_ (m^2^ g^−1^)	*S*_meso_/*S*_BET_ (%)	Pore Volume (cm^3^ g^−1^)	Average Pore Size (nm)
RH-1600	121.9	83.5	0.278	9.1
SiC-C@Fe_3_O_4_	125.5	83.6	0.327	10.4

**Table 3:** Electrocatalytic parameters of SiC-C@Fe_3_O_4_ composites compared with other samples.

Material	Electrolyte	Current Density (mA cm^−2^)	Overpotential (mV)	Tafel Slope (mV dec^−1^)	Reference
Fe_3_O_4_/NCMTs-800(IL)	1.0 M KOH	10	310	80.81	[57]
Fe_20_@N/HCSs	1.0 M KOH	10	289	52.4	[50]
H-Co_9_S_8_/ Fe_3_O_4_@SNC	0.1 M KOH	10	280	87	[58]
Fe_3_O_4_/CoO CNTs	1.0 M KOH	10	270	59	[59]
SiC-C@Fe_3_O_4_	1.0 M KOH	10	260	56.93	Present study

## Data Availability

The data presented in this study are available on request from the corresponding author.

## Sample Availability

Samples of the compounds Si-C@Fe_3_O_4_ are available from the authors.

## References

[1] You M., Du X., Hou X., Wang Z., Zhou Y., Ji H., Zhang L., Zhang Z., Yi S., Chen D. (2022). In-situ growth of ruthenium-based nanostructure on carbon cloth for superior electrocatalytic activity towards HER and OER. Appl. Catal. B Environ..

[2] Feng Y., Zhao Z., Li F., Bu L., Shao Q., Li L., Wu J., Zhu X., Lu G., Huang X. (2021). Highly Surface-Distorted Pt Superstructures for Multifunctional Electrocatalysis. Nano Lett..

[3] Paudel D.R., Pan U.N., Singh T.I., Gudal C.C., Kim N.H., Lee J.H. (2021). Fe and P Doped 1T-Phase Enriched WS_2_3D-Dendritic Nanostructures for Efficient Overall Water Splitting. Appl. Catal. B Environ..

[4] Sondermann L., Jiang W., Shviro M., Spieß A., Woschko D., Rademacher L., Janiak C. (2022). Nickel-Based Metal-Organic Frameworks as Electrocatalysts for the Oxygen Evolution Reaction (OER). Molecules.

[5] Li C., Li T., Yu G., Chen W. (2023). Theoretical Investigation of HER and OER Electrocatalysts Based on the 2D R-graphyne Completely Composed of Anti-Aromatic Carbon Rings. Molecules.

[6] Ejeta S.Y., Imae T. (2022). Cobalt Incorporated Graphitic Carbon Nitride as a Bifunctional Catalyst for Electrochemical Water-Splitting Reactions in Acidic Media. Molecules.

[7] Yan H., Wang X., Linkov V., Ji S., Wang R. (2023). Selectivity of Oxygen Evolution Reaction on Carbon Cloth-Supported δ-MnO_2_ Nanosheets in Electrolysis of Real Seawater. Molecules.

[8] Sato Y., Yamada N., Kitano S., Kowalski D., Aoki Y., Habazaki H. (2022). High-corrosion-resistance mechanism of graphitized platelet-type carbon nanofibers in the OER in a concentrated alkaline electrolyte. J. Mater. Chem. A.

[9] Singh H., Marley-Hines M., Chakravarty S., Nath M. (2022). Multi-walled carbon nanotube supported manganese selenide as a highly active bifunctional OER and ORR electrocatalyst. J. Mater. Chem. A.

[10] Hou G., Jia X., Kang H., Qiao X., Liu Y., Li Y., Wu X., Qin W. (2022). CoNi nano-alloys modified yolk-shell structure carbon cage via Saccharomycetes as carbon template for efficient oxygen evolution reaction. Appl. Catal. B Environ..

[11] Chen W., Xie Y., Gao X., Li L., Lin Z. (2022). Simultaneous optimization of CoIr alloy nanoparticles and 2D graphitic-N doped carbon support in CoIr@CN by Ir doping for enhanced oxygen and hydrogen evolution reactions. J. Mater. Chem. A.

[12] Quílez-Bermejo J., García-Dalí S., Daouli A., Zitolo A., Canevesi R.L.S., Emo M., Izquierdo M.T., Badawi M., Celzard A., Fierro V. (2023). Advanced Design of Metal Nanoclusters and Single Atoms Embedded in C_1_N_1_-Derived Carbon Materials for ORR, HER, and OER. Adv. Funct. Mater..

[13] Zhang M., Li H., Chen J., Ma F.-X., Zhen L., Wen Z., Xu C.-Y. (2022). Transition Metal (Co, Ni, Fe, Cu) Single-Atom Catalysts Anchored on 3D Nitrogen-Doped Porous Carbon Nanosheets as Efficient Oxygen Reduction Electrocatalysts for Zn–Air Battery. Small.

[14] Ma L., Wei Z., Zhao C., Meng X., Zhang H., Song M., Wang Y., Li B., Huang X., Xu C. (2023). Hierarchical superhydrophilic/superaerophobic 3D porous trimetallic (Fe, Co, Ni) spinel/carbon/nickel foam for boosting oxygen evolution reaction. Appl. Catal. B Environ..

[15] Lu L., Zhang Y., Chen Z., Feng F., Teng K., Zhang S., Zhuang J., An Q. (2022). Synergistic promotion of HER and OER by alloying ternary Zn-Co-Ni nanoparticles in N-doped carbon interfacial structures. Chin. J. Catal..

[16] Feng Y., Jiang J., Xu Y., Wang S., An W., Chai Q., Prova U.H., Wang C., Huang G. (2023). Biomass derived diverse carbon nanostructure for electrocatalysis, energy conversion and storage. Carbon.

[17] Zhang X., Han R., Liu Y., Li H., Shi W., Yan X., Zhao X., Li Y., Liu B. (2023). Porous and graphitic structure optimization of biomass-based carbon materials from 0D to 3D for supercapacitors: A review. Chem. Eng. J..

[18] Queneau Y., Han B. (2022). Biomass: Renewable carbon resource for chemical and energy industry. Innovation.

[19] Tian P., Zhan G., Tian J., Tan K.B., Guo M., Han Y., Fu T., Huang J., Li Q. (2022). Direct CO_2_ hydrogenation to light olefins over ZnZrOx mixed with hierarchically hollow SAPO-34 with rice husk as green silicon source and template. Appl. Catal. B Environ..

[20] Dafiqurrohman H., Safitri K.A., Setyawan M.I.B., Surjosatyo A., Aziz M. (2022). Gasification of rice wastes toward green and sustainable energy production: A review. J. Clean. Prod..

[21] Lan K., Zhao D. (2022). Functional Ordered Mesoporous Materials: Present and Future. Nano Lett..

[22] Stachowicz L., Singh S.K., Wender E., Girshick S.L. (1993). Synthesis of ultrafine SiC from rice hulls (husks): A plasma process. Plasma Chem. Plasma Process..

[23] Zhao Z., Xie H., Qu J., Zhao H., Ma Q., Xing P., Song Q., Wang D., Yin H. (2019). A Natural Transporter of Silicon and Carbon: Conversion of Rice Husks to Silicon Carbide or Carbon-Silicon Hybrid for Lithium-Ion Battery Anodes via a Molten Salt Electrolysis Approach. Batter. Supercaps.

[24] Qian J.-M., Wang J.-P., Qiao G.-J., Jin Z.-H. (2004). Preparation of porous SiC ceramic with a woodlike microstructure by sol-gel and carbothermal reduction processing. J. Eur. Ceram. Soc..

[25] Yang S., Kiraly B., Wang W.Y., Shang S., Cao B., Zeng H., Zhao Y., Li W., Liu Z.-K., Cai W. (2012). Fabrication and Characterization of Beaded SiC Quantum Rings with Anomalous Red Spectral Shift. Adv. Mater..

[26] Satapathy L.N., Ramesh P.D., Agrawal D., Roy R. (2005). Microwave synthesis of phase-pure, fine silicon carbide powder. Mater. Res. Bull..

[27] Singh B., Siddique R., Cachim P. (2018). 13—Rice husk ash. Waste and Supplementary Cementitious Materials in Concrete.

[28] Li D., Zhang X., Wang Y., Yan X., Zong P., Lu G., Tian Y. (2021). Activation of rice hull char with steam to improve lithium storage performance of SiO2/C. J. Anal. Appl. Pyrolysis.

[29] Han H.-W., Liu H.-S. (1999). Characterization of vapour deposited products in furnace tube during SiC synthesis from carbonized rice hulls. Ceram. Int..

[30] Temeche E., Yu M., Laine R.M. (2020). Silica depleted rice hull ash (SDRHA), an agricultural waste, as a high-performance hybrid lithium-ion capacitor. Green Chem..

[31] Yu M., Temeche E., Indris S., Laine R.M. (2021). Adjusting SiO_2_ : C mole ratios in rice hull ash (RHA) to control carbothermal reduction to nanostructured SiC, Si_3_N_4_ or Si_2_N_2_O composites. Green Chem..

[32] Krishnarao R.V., Mahajan Y.R. (1996). Formation of SiC whiskers from raw rice husks in argon atmosphere. Ceram. Int..

[33] Alweendo S.T., Johnson O.T., Shongwe M.B., Kavishe F.P.L., Borode J.O. (2019). Synthesis, Optimization and Characterization of Silicon Carbide (SiC) from Rice Husk. Procedia Manuf..

[34] Nutt S.R. (1988). Microstructure and Growth Model for Rice-Hull-Derived SiC Whiskers. J. Am. Ceram. Soc..

[35] Niyomwas S. (2017). Synthesis and characterization of silicon-silicon carbide composites from rice husk ash via self-propagating high temperature synthesis. J. Met. Mater. Miner..

[36] Li J., Chen C., Lv Z., Ma W., Wang M., Li Q., Dang J. (2023). Constructing heterostructures of ZIF-67 derived C, N doped Co_2_P and Ti_2_VC_2_T_x_ MXene for enhanced OER. J. Mater. Sci. Technol..

[37] Yin H.L., Yang W., Zhao L.C., Hu X.M., Liu S.Q., Cui C.X., Wang X. (2022). Fabrication and mechanical property of three-dimensional carbon fiber reinforced Mg-based bulk metallic glass matrix composite. Mater. Sci. Eng. A.

[38] Yin H.L., Liu S.Q., Zhao L.C., Cui C.X., Wang X. (2021). Vacuum infiltration molding and mechanical property of short carbon fiber reinforced Ti-based metallic glass matrix composite. J. Mater. Process. Technol..

[39] Gong S., Sun Y., Zheng K., Jiang G., Li L., Feng J. (2020). Degradation of levofloxacin in aqueous solution by non-thermal plasma combined with Ag_3_PO_4_/activated carbon fibers: Mechanism and degradation pathways. Sep. Purif. Technol..

[40] Jia Y., Sun X., Shi Z., Jiang K., Liu H., Ben J., Li D. (2018). Modulating the Surface State of SiC to Control Carrier Transport in Graphene/SiC. Small.

[41] Zhou F., Gan M., Yan D., Chen X., Peng X. (2023). Hydrogen-Rich Pyrolysis from Ni-Fe Heterometallic Schiff Base Centrosymmetric Cluster Facilitates NiFe Alloy for Efficient OER Electrocatalysts. Small.

[42] Janghorban K., Tazesh H.R. (1999). Effect of catalyst and process parameters on the production of silicon carbide from rice hulls. Ceram. Int..

[43] Weng W., Wang S., Xiao W., Lou X.W. (2020). Direct Conversion of Rice Husks to Nanostructured SiC/C for CO_2_ Photoreduction. Adv. Mater..

[44] Freitas J.C.C., Moreira J.S., Emmerich F.G., Bonagamba T.J. (2004). Development of Si/C/N/O ceramics from pyrolyzed and heat-treated rice hulls. J. Non-Cryst. Solids.

[45] Li G., Tang Y., Fu T., Xiang Y., Xiong Z., Si Y., Guo C., Jiang Z. (2022). S, N co-doped carbon nanotubes coupled with CoFe nanoparticles as an efficient bifunctional ORR/OER electrocatalyst for rechargeable Zn-air batteries. Chem. Eng. J..

[46] Lamy-Mendes A., Malfait W.J., Sadeghpour A., Girão A.V., Silva R.F., Durães L. (2021). Influence of 1D and 2D carbon nanostructures in silica-based aerogels. Carbon.

[47] Li C., Chen H., Zhang L., Jiao S., Zhang H., Zhang J., Li P., Tao Y., Zhao X. (2021). Rice Hull-Derived Carbon for Supercapacitors: Towards Sustainable Silicon-Carbon Supercapacitors. Polymers.

[48] Zhao X., Li C., Sha L., Yang K., Gao M., Chen H., Jiang J. (2022). In-Built Fabrication of MOF Assimilated Porous Hollow Carbon from Pre-Hydrolysate for Supercapacitor. Polymers.

[49] Jiao S., Zhang L., Li C., Zhang H., Zhang J., Li P., Tao Y., Zhao X., Chen H., Jiang J. (2022). Efficient construction of a carbon-based symmetric supercapacitor from soybean straw by coupling multi-stage carbonization and mild activation. Ind. Crops Prod..

[50] Wang B., Ye Y., Xu L., Quan Y., Wei W., Zhu W., Li H., Xia J. (2020). Space-Confined Yolk-Shell Construction of Fe_3_O_4_ Nanoparticles Inside N-Doped Hollow Mesoporous Carbon Spheres as Bifunctional Electrocatalysts for Long-Term Rechargeable Zinc–Air Batteries. Adv. Funct. Mater..

[51] Bianchetti E., Perilli D., Di Valentin C. (2023). Improving the Oxygen Evolution Reaction on Fe_3_O_4_(001) with Single-Atom Catalysts. ACS Catal..

[52] Xu W., Zhong W., Yang C., Zhao R., Wu J., Li X., Yang N. (2022). Tailoring interfacial electron redistribution of Ni/Fe_3_O_4_ electrocatalysts for superior overall water splitting. J. Energy Chem..

[53] Huang W., Peng C., Tang J., Diao F., Nulati Yesibolati M., Sun H., Engelbrekt C., Zhang J., Xiao X., Mølhave K.S. (2022). Electronic structure modulation with ultrafine Fe_3_O_4_ nanoparticles on 2D Ni-based metal-organic framework layers for enhanced oxygen evolution reaction. J. Energy Chem..

[54] Royer L., Bonnefont A., Asset T., Rotonnelli B., Velasco-Vélez J.-J., Holdcroft S., Hettler S., Arenal R., Pichon B., Savinova E. (2023). Cooperative Redox Transitions Drive Electrocatalysis of the Oxygen Evolution Reaction on Cobalt–Iron Core–Shell Nanoparticles. ACS Catal..

[55] Liu H.-J., Zhang S., Zhou Y.-N., Yu W.-L., Ma Y., Wang S.-T., Chai Y.-M., Dong B. (2023). Dynamically Stabilized Electronic Regulation and Electrochemical Reconstruction in Co and S Atomic Pair Doped Fe_3_O_4_ for Water Oxidation. Small.

[56] Luo H., Zhao X., Zhang T., Si R., Gong X., Li C., Kong F., Liu Y., Jiang J., Chen H. (2023). Self-supporting porous wood-based carbon with metal-organic framework derived metal bridges for effectively electrocatalytic hydrogen evolution at large current density. Int. J. Hydrogen Energy.

[57] Liu G., Wang B., Ding P., Ye Y., Wei W., Zhu W., Xu L., Xia J., Li H. (2019). Reactable ionic liquid in situ-induced synthesis of Fe_3_O_4_ nanoparticles modified N-doped hollow porous carbon microtubes for boosting multifunctional electrocatalytic activity. J. Alloys Compd..

[58] Gan L., Fang J., Wang M., Hu L., Zhang K., Lai Y., Li J. (2018). Preparation of double-shell Co_9_S_8_/Fe_3_O_4_ embedded in S/N co-decorated hollow carbon nanoellipsoid derived from Bi-Metal organic frameworks for oxygen evolution reaction. J. Power Sources.

[59] Luo Y., Yang H., Ma P., Luo S., Zhao Z., Ma J. (2020). Fe_3_O_4_/CoO Interfacial Nanostructure Supported on Carbon Nanotubes as a Highly Efficient Electrocatalyst for Oxygen Evolution Reaction. ACS Sustain. Chem. Eng..

